# Classifying Image Stacks of Specular Silicon Wafer Back Surface Regions: Performance Comparison of CNNs and SVMs

**DOI:** 10.3390/s19092056

**Published:** 2019-05-02

**Authors:** Corinna Kofler, Robert Muhr, Gunter Spöck

**Affiliations:** 1 Department of Statistics, Universität Klagenfurt, 9020 Klagenfurt, Austria; gunter.spoeck@aau.at; 2 Department of Process Stability, Infineon Technologies Austria AG, 9500 Villach, Austria; robert.muhr@infineon.com

**Keywords:** silicon wafer, image classification, convolutional neural network, support vector machine

## Abstract

In this work, we compare the performance of convolutional neural networks and support vector machines for classifying image stacks of specular silicon wafer back surfaces. In these image stacks, we can identify structures typically originating from replicas of chip structures or from grinding artifacts such as comets or grinding grooves. However, defects like star cracks are also visible in those images. To classify these image stacks, we test and compare three different approaches. In the first approach, we train a convolutional neural net performing feature extraction and classification. In the second approach, we manually extract features of the images and use these features to train support vector machines. In the third approach, we skip the classification layers of the convolutional neural networks and use features extracted from different network layers to train support vector machines. Comparing these three approaches shows that all yield an accuracy value above 90%. With a quadratic support vector machine trained on features extracted from a convolutional network layer we achieve the best compromise between precision and recall rate of the class star crack with 99.3% and 98.6%, respectively.

## 1. Introduction

Wafers affected by star cracks are discarded in a semiconductor production, since such wafers can break once exposed to further stress in subsequent process steps. A wafer breakage during a process step causes downtime of fabrication tools due to laborious cleaning procedures. To avoid such wafer breakages, wafers affected by star cracks are currently sorted out by human visual inspections. Automatically inspecting these wafer back surfaces is challenging since they are specular and thus virtually invisible. Yet, three-dimensional defects such as star cracks are observable by the disturbances they cause in mirror images of regular patterns. This principle is applied in deflectometric measurement methods [1]. Such deflectometric inspection systems have been introduced to the semiconductor industry, recently. They provide promising images of whole wafer back surfaces and approaches classifying the visible three-dimensional structures in these images with statistical pattern recognition are available. In a first work we demonstrated that classifying defects in topography images of silicon wafers is feasible [2]. We induced defects in a controlled manner on raw silicon wafers and classified these defects with selected classifiers based on extracted features from the topography images. In a second work, we developed a pattern recognition algorithm for topography images of productive wafers [3]. In topography images of productive wafers replicas of chip structures as well as grinding artifacts such as grinding grooves and comets can be visible. To distinguish between replicas of chip structures, grinding grooves, and comets and to separate them from star cracks we developed a pattern recognition algorithm consisting of cascaded support vector machines. However, addressing this task with deep learning yet must be investigated.

Deep convolutional networks are well suited for image recognition tasks and have brought breakthroughs in this domain [4]. Convolutional neural networks automatically extract features and classify them. Therefore, human domain experts for manual feature engineering are not required. Raw image data are fed into the convolutional neural network and each network layer learns features by representation learning. This behavior allows convolutional neural networks to learn the features from the data, which is the reason they perform well for image recognition tasks. Hence, such convolutional neural networks are used in various application fields for defect detection [5,6,7,8,9,10,11,12]. More information regarding deep neural networks can be found in [13].

In this paper, we compare the performance of simple convolutional neural networks (CNNs) and support vector machines (SVMs) for classifying our image stacks. In Section 2 we describe our image stacks in detail and divide them into a training set and a test set. Then we describe the applied classification approaches in detail. We start with the simple CNNs, followed by the SVMs trained on manually extracted and selected features, and we end with the SVMs trained on features extracted from different layers of the simple CNNs. In Section 3 we first present the classification results of the applied approaches in the same order. Then we compare and discuss the results of the different models. Finally, we draw a conclusion in Section 4.

## 2. Material and Methods

### 2.1. Image Stacks

We have image stacks of specular silicon wafer back surface regions acquired by a deflectometric inspection system. In these image stacks, we can identify three-dimensional structures. They typically originate from replicas of chip structures and we consider them as background. Besides, we see structures caused by grinding artifacts such as comets or grinding grooves. However, most importantly for us, also star cracks cause visible signatures in those image stacks. Hence, we can group these image stacks into four classes: Background, comet, grinding grove, and star crack. Therefore, we can detect star cracks and monitor grinding artifacts.

**Figure 1:**
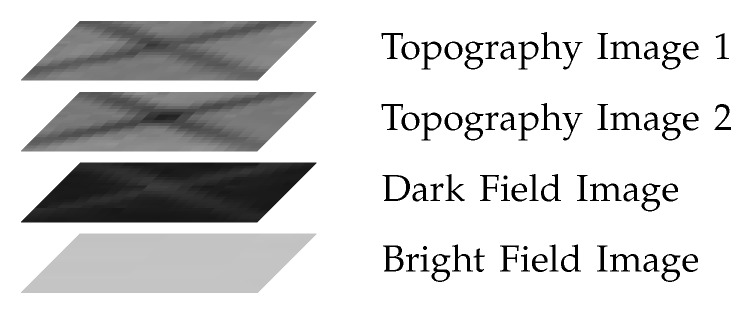
Image stack of a star crack as an example.

Each image stack consists of four images showing the exact same region of the wafer back surface. The images have a size of 21 pixels × 21 pixels. Figure 1 shows an image stack containing a star crack as an example. From the top to the bottom we have two topography images, a dark field image, and a bright field image.

**Figure 2:**
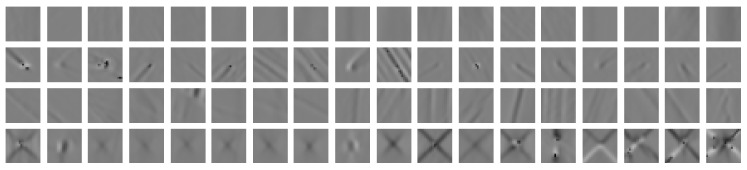
Topography image examples randomly selected from our sample set.

Most of the information for detecting star cracks is visible in the topography images. Hence, we start with analyzing the topography images in detail. Figure 2 shows 18 randomly selected examples per class of our sample set. The first row shows background regions. The second and third rows show comet and grinding groove regions, respectively. The last row shows star crack regions. Analyzing these sample images reveals that background regions and grinding groove regions might be difficult to distinguish. Additionally, we observe that some comet regions look similar to star crack regions. Lastly, we see that the star crack signals vary in size, shape, and contrast.

In total we have 2300 image stacks evenly distributed among our four classes. We randomly divide the sample set into 75% training samples and 25% test samples. We will use the exact same split for our performance comparisons. Hence, we will use the same training samples to train the CNNs and the SVMs as well as the same test samples for testing the different trained models.

### 2.2. Classification

We classify our image stacks with three different approaches. In the first approach we create a simple convolutional neural network architecture and train different nets. In the second approach we manually extract features out of the image stacks and train with them SVMs. In the third approach we use the trained simple CNNs as feature extractors. With the features obtained from the different network layers we then train SVMs. We compare the three approaches by the overall accuracy and the precision and recall rate of the class star crack, respectively. More information regarding classifier performance measures can be found in [14,15].

#### 2.2.1. CNN

CNNs for deep learning are used for image recognition tasks performing feature extraction and classification. The features are extracted by the convolutional layers filtering the image. The filter weights are learned during the training. The pooling layers reduce the size of the image feature descriptors preserving useful image features. Based on these image features the fully connected layer and the SoftMax layer classify the input images. Several pre-trained CNNs such as the GoogLeNet, the VGG-16, or the AlexNet are available for image classification. They are trained on more than a million images from the ImageNet database [16]. These pre-trained nets can classify images into 1000 different classes. All of them require 3 channel RGB input images with a size of around 225 pixels × 225 pixels. Our input images are too small with a size of just 21 pixels × 21 pixels and contain fewer features, therefore not suited for complex CNNs. However, several publications show that simple convolutional neural network architectures are suited for classifying these images as for example in [17,18,19].

**Figure 3:**
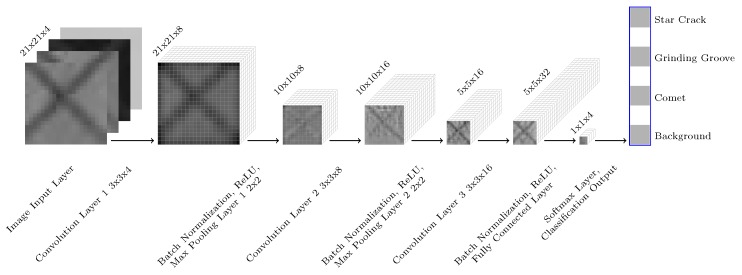
Simple convolutional neural network architecture for classifying our 21×21×4 image stacks of wafer back surface regions.

Accordingly, we define a simple convolutional neural network architecture (Figure 3) on our own and train it from scratch on our image stacks. This simple convolutional neural network consists of 15 layers. The image input layer specifies the input image size with 21 pixels × 21 pixels and 4 channels. Then we have three convolutional layers each followed by a batch normalization layer and a nonlinear activation function layer. The first convolutional layer consists of 8 filters, the second one of 16 filters, and the third one of 32 filters. All filters have a kernel size of 3 pixels × 3 pixels. For each convolutional layer, the stride and the padding is set to ‘1’. The batch normalization layers between each convolutional layer and the following nonlinear activation function layer reduce the sensitivity to the network initialization and speed up the training. For the nonlinear activation function layers, we use rectified linear units (ReLU). The first two convolutional layers additionally have a max pooling layer after the ReLU layers. Max pooling layers remove redundant information and reduce the spatial size of the feature maps. They return the maximum value of a 2 pixels × 2 pixels region. For each max pooling layer, the stride is set to ‘2’ and the padding is set to ‘0’. Hence, the feature map is reduced to 10 pixels × 10 pixels by the first max pooling layer and the second one reduces the spatial size to 5 pixels × 5 pixels. In our network, the convolutional and down-sampling layers are followed by one fully connected layer. This fully connected layer combines all the features learned by the previous layers to assign the images to one of our four classes. The output of this classification layer is normalized by the subsequent SoftMax activation function. The output of this SoftMax activation function are positive values summing up to one, which are used as classification probabilities. These classification probabilities are used by the final classification layer to assign the input to one of the four classes: Background, comet, grinding groove, or star crack.

We train this simple convolutional neural network using a stochastic gradient descent with momentum (SGDM) and a suitable fixed learning rate and a suitable number of maximum epochs. An epoch is a full training cycle on all training samples and the training samples are shuffled every epoch. In our case each epoch consists of 12 iterations. An iteration corresponds to the mini-batch size, which is set to 140 samples in our case. It is a subset of the training samples used to evaluate the gradient of the loss function and update the weights. Every 6th iteration we test our network with the test samples. We define the network stopping criteria with a validation patience of 5. Which means that the training stops once the loss on the validation samples is five times in a row larger or equal than the previously smallest loss. The test samples are not used to update the network weights. We repeat this training hundred times to analyze the variances of the training and the testing accuracies.

#### 2.2.2. SVM

SVMs have good performance in dealing with classification problems [20]. Accordingly, we choose to use SVMs in this work. We train SVMs with a linear, a quadratic, a cubic, and a medium Gaussian kernel. To train SVMs a set of image features is required, hence we start with feature extraction as described in [21]. We manually extract texture features and region-based features out of the image stacks, as well as the histogram values divided into 16 bins:

(1) $$\begin{array}{c} H\left(k\right)=card\{X|B_{k}\;\leq \;I\left(X\right)\;<\;B_{k+1}\},\;k=\left\{1,2,3,...,16\right\},B=\left\{0,16,32,...,256\right\} \end{array}$$

where *B* is the break vector, *k* the histogram bin, I(X) is the intensity value at column *X*, and *X* is the column number of the intensity vector. The intensity histogram provides information about the intensity distribution of our images as described in ([22], p. 120ff). Additionally, we calculate the empirical isotropic semivariogram:

(2) $$\begin{array}{c} \gamma \left(h\right)=\frac{1}{2N(h)}\sum_{\left(s_{i},s_{j}\right)\in N\left(h\right)}{\left(z\left(s_{i}\right)-z\left(s_{j}\right)\right)}^{2},\;h=s_{i}-s_{j} \end{array}$$

where *h* is the specified distance range, N(h) is the number of data pairs falling within the specified distance range, $s_{i}$, $s_{j}$ are the two locations of data points, $z(s_{i})$, $z(s_{j})$ are the intensity values of the two data points, and γ(h) is the semivariogram value for the specified distance range. With this empirical isotropic semivariogram we describe the roughness of our images [23].

To extract the region-based features we binarize the topography images with two thresholds $T_{1}=100$ and $T_{2}=150$ and extract the regions properties of the largest object in the binary image. A pixel $p_{\left(x,y\right)}$ belongs to the background if $T_{1}\;\leq \;I_{\left(x,y\right)}\;\leq \;T_{2}$ and is set to the value ‘0’. A pixel $p_{\left(x,y\right)}$ belongs to the foreground if $I\left(x,y\right)<T_{1}$ or $I\left(x,y\right)>T_{2}$ and is set to the value ‘1’ representing the object.

Finally, we add all extracted features of each image stack to a one-dimensional feature vector, each consisting of 232 features and save them in a data matrix. Subsequently, we standardize the data matrix using *z*-scores:

(3) $$Z=\frac{X-\mu }{\sigma }$$

where *X* is the data vector containing the data values, μ is the arithmetic mean, and σ the standard deviation of the data vector *X*.

Furthermore, we reduce the dimension of our feature vectors to increase the classification performance by eliminating redundant and irrelevant features. More information about feature selection can be found in [24,25,26]. Generally, there are two feature selection methods available, feature extraction and feature selection. Feature extraction methods, such as the principal component analysis transform the whole feature space retaining underlying uncorrelated components. In contrast, feature selection methods search for the most discriminating features among all features and thus identify irrelevant features. Therefore, the number of features to be calculated from images can be reduced, which saves computation time. Hence, we choose to apply a feature selection method. We apply the neighborhood component feature selection [27], since it is distribution independent and therefore applicable to our non-normally distributed feature vectors. The neighborhood component feature selection learns the features weights by maximizing the expected classification accuracy with a regularization term. Hence, this feature selection method calculates the feature weights based on the performance of a nearest neighbor classifier. However, since the importance of features can often be generalized in practice, we use its selected features to train SVMs.

#### 2.2.3. CNN Features and SVM

CNNs can be used to extract features out of images, which in turn can be used to train statistical classifiers. The first convolutional layer looking at the raw input images learns basic features such as where the edges are in the images. The deeper layers in the network combine the information from the previous layers and form higher-level image features. These higher-level image features can be extracted and used to train a statistical classifier. This is of advantage if a pre-trained net such as the AlexNet can be applied on a new set of images. If the number of new image samples is rather small, transfer learning might result in a lower performance compared to using the high-level features of the net in combination with a statistical classifier. Additionally, the training time is shorter. For our small 21 × 21 × 4 image stacks there is no pre-trained net available, as already discussed. However, we extract the features at different layers of our trained simple convolutional neural net and train SVMs to compare their performances with the SVMs trained on our manually extracted features.

## 3. Results and Discussion

To compare the performance of the three different approaches, we train the simple convolutional neural network architecture hundred times and cross-validate the SVMs using 10 × 10 cross-validation. We use the exact same training samples for training the simple CNNs and cross-validating the SVMs. Finally, we compare the performance of the different trained models on the test samples.

### 3.1. CNN

**Figure 4:**
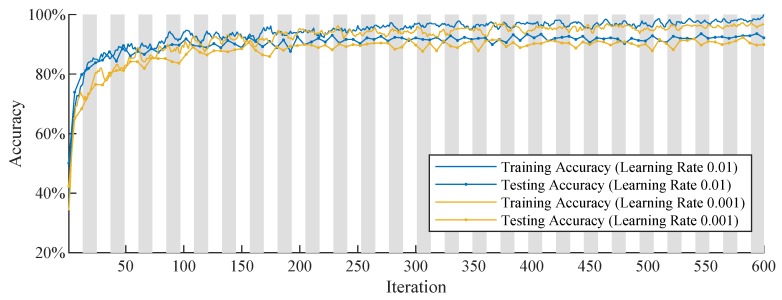
Training progress plot of the convolutional neural network with different learning rates.

To determine a suitable learning rate as well as the maximum number of epochs, we train the simple convolutional neural network with different learning rates over 50 Epochs in the first step. Overall, we compare the training progress plots with learning rates of 0.1, 0.01, 0.001 and 0.0001. However, to present a clear graphic, Figure 4 only compares the best two training progress plots with a learning rate of 0.01 and a learning rate of 0.001. The training and testing accuracies are depicted on the y-axis. The training accuracies are smoothed with a moving average filter with a span of 5. The x-axis shows the number of iterations. The background stripe pattern in the plot marks the different epochs. From this plot we derive that we get a slightly better performance with a learning rate of 0.01 and that we can set the maximum number of epochs to 20, since testing accuracy improvement is minor from around the 14th epoch onward.

**Figure 5:**
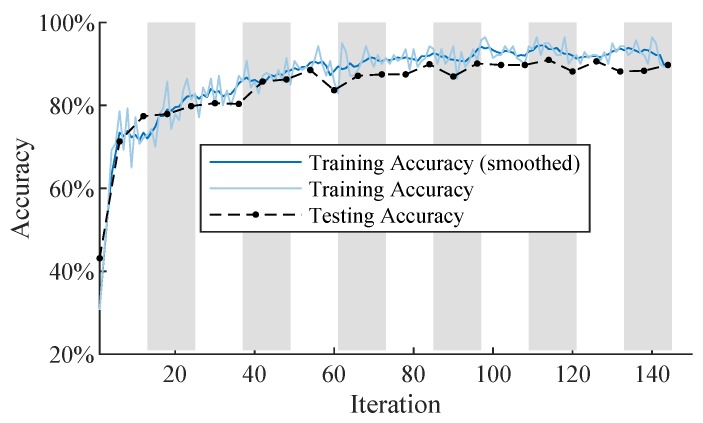
Training progress plot of the convolutional neural network.

Hence, we continue to train the simple convolutional neural network once, with the fixed learning rate of 0.01 and a maximum number of 20 epochs. Additionally, we define the network stopping criteria with a validation patience of 5. The training progress plot (Figure 5) shows the training and testing accuracy on the y-axis. Additionally, we plot the smoothed training accuracy applying a moving average filter with a span of 5. The x-axis shows the number of iterations. The background stripe pattern in the plot marks the different epochs. The plot shows that the stopping criterion is met after 144 iterations achieving a testing accuracy of 89.7%. The total training takes around 90 s on a single i5 CPU using MATLAB [28].

**Figure 6:**
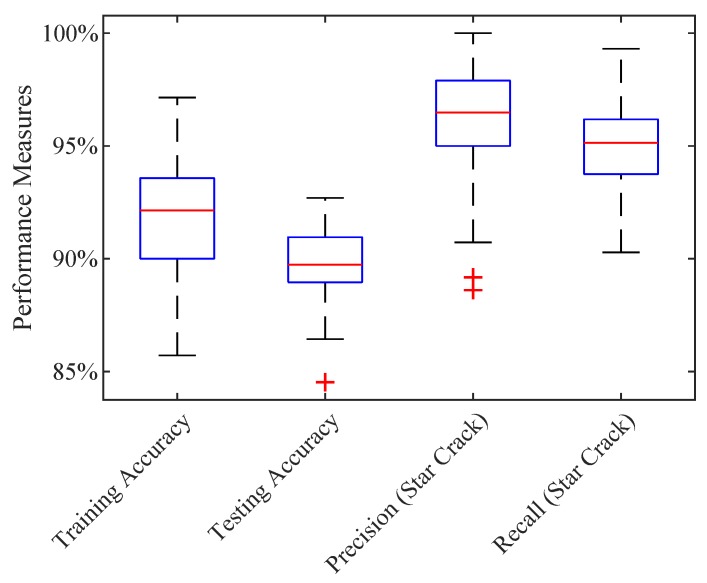
Performance measures of 100 times trained the simple CNN.

Due to the short training time, we can repeat the training hundred times to obtain the distribution of the classification performance. The boxplot (Figure 6) shows from the left to the right the training accuracy, the testing accuracy, and the testing precision and the testing recall of the class star crack on the x-axis. The y-axis depicts the performance measures in percent. This plot shows that our first training attempt, reaching a testing accuracy of 89.7%, lies around the median. Consequently, much worse or better results are possible. Furthermore, the plot shows that the precision and the recall of the class star crack are better than the overall testing accuracy. This is important for us, since we focus on correctly detecting all image stacks containing a star crack.

**Figure 7:**
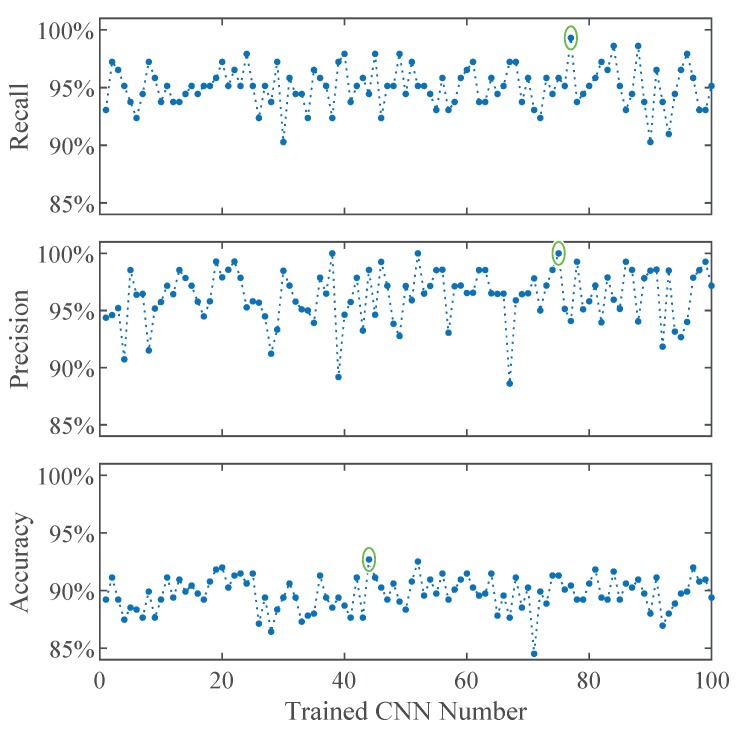
Performances of 100 times trained the simple CNN.

**Figure 8:**
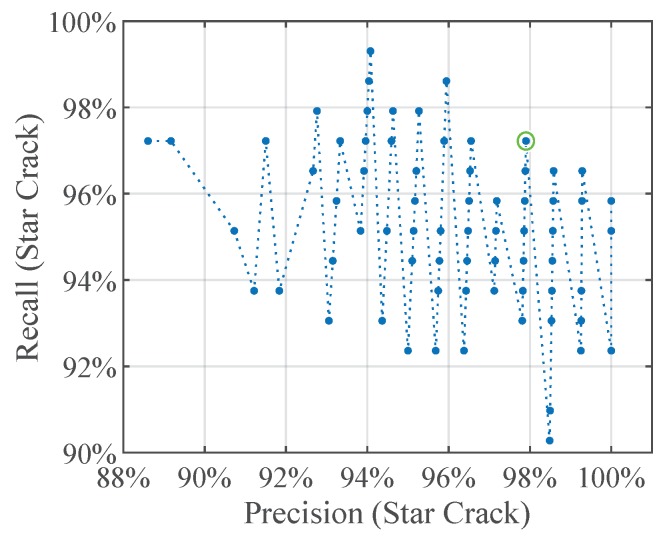
CNNs: Recall vs. precision of the class star crack.

For the classifier comparison we select four of the trained simple CNNs for further comparisons in this work. The net with the best precision, the one with the best recall, and the net with the best compromise between precision and recall of the class star crack, as well as the net with the best overall accuracy. Figure 7 shows the performance measures plotted on the y-axes in percent and the number of the trained convolutional neural network on the x-axes. The lower boxplot is generated from the testing accuracy of the trained CNNs and the circle marks the net ‘CNN 44’ which has the best overall testing accuracy. The middle plot as well as the upper plot show the precision and recall rates of the class star crack, respectively. The nets ‘CNN 75’ with the best precision and the net ‘CNN 77’ with the best recall of the class star crack are marked with circles too. The net ‘CNN 20’ with the best compromise between precision and recall of the class star crack is marked with a circle in Figure 8, which plots the precision against the recall rate of the class star crack.

### 3.2. SVM

Training SVMs requires preceding feature extraction. We extract texture and region-based features from the training as well as the test image stacks, using the same split as for training the CNNs. In total we extract 232 features of each image stack and write them into data set tables. The training data set contains all extracted features and the corresponding label of the training image stacks. The test data set contains all extracted features and the corresponding label of the test image stacks.

**Figure 9:**
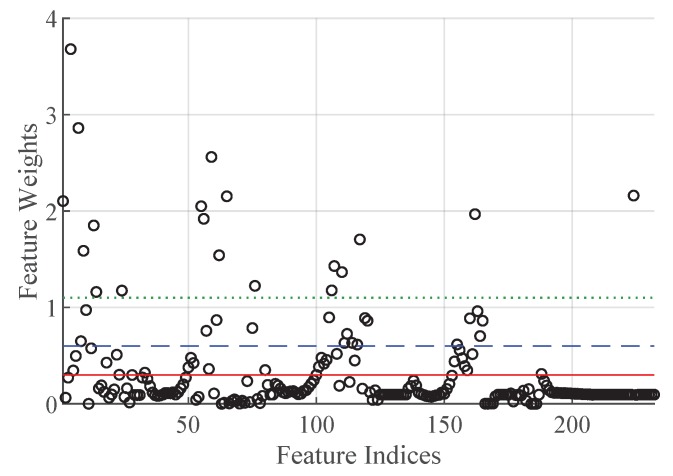
Plot of the neighborhood component feature selection.

The following feature selection reduces the dimensionality of our data set. We apply the neighborhood component feature selection only on our training data set. Figure 9 shows the assigned weights of the 232 extracted features. The feature indexes are depicted on the x-axes and the corresponding feature weights on the y-axes. The horizontal lines mark the three different feature subsets which we use to train the SVMs. For each feature subset the features below the corresponding horizontal line are neglected. The dotted line indicates the feature weight threshold 1.1, the dashed line the threshold 0.6, and the solid line the threshold 0.3. We determine these feature weight thresholds based on the cross-validation performances of our target support vector machine with a quadratic kernel. A preceding 10-by-10 repeated cross-validation comparison of SVMs with a linear, a quadratic, a cubic, and a Gaussian kernel revealed that the one with the quadratic kernel shows the best performance.

**Figure 10:**
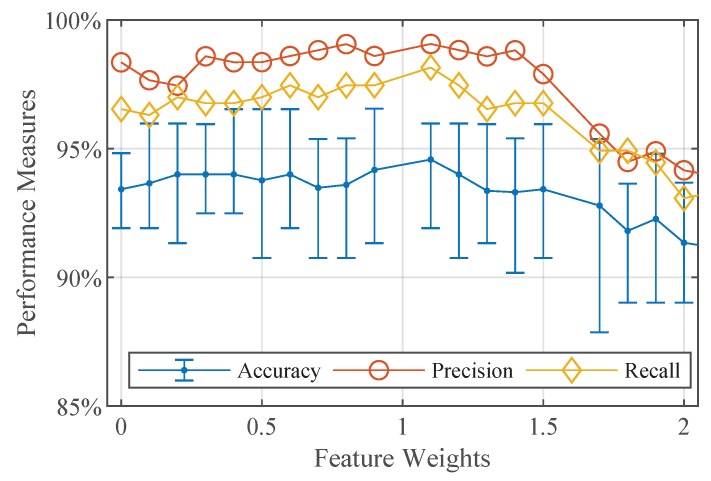
Quadratic SVMs cross-validated on different feature sets.

For selecting the feature weight thresholds, we analyze the performance curves of the quadratic support vector machine using 10-fold cross-validation (Figure 10). The mean performance measures of the classifier are plotted on the y-axis. For the overall accuracy curve, we additionally depict the error bars. The x-axis shows the features sorted in descending order according to their weights derived from the neighborhood component feature selection. We start with cross-validating the quadratic support vector machine with all extracted features and neglect step by step features below a certain weight with a step size of 0.1. Analyzing the obtained performance curves let us identify the feature weight threshold of 1.1 for the first feature subset. With this feature sub set we achieve the highest accuracy and the highest precision and recall of the class star crack. Additionally, we select the feature weight threshold of 0.6 for the second feature subset and the feature weight threshold 0.3 for the third feature subset. The first feature subset consists of 19 selected features, the second one of 36 selected features, and the third one of 62 selected features. We use these selected feature subsets to train quadratic SVMs and compare their performance.

**Figure 11:**
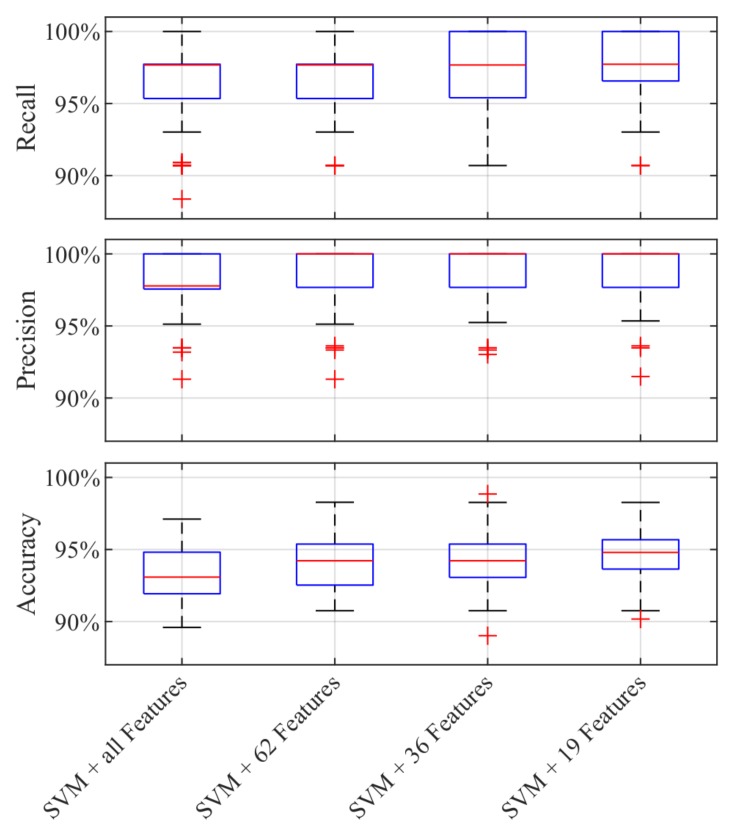
10-by-10 cross-validation results of SVMs trained on different extracted feature sets.

To compare the performances of the quadratic SVMs trained on the different feature sub sets in detail, we perform a 10-by-10 repeated cross-validation for each sub set (Figure 11). The x-axes show the tested quadratic SVMs with the different feature sets. The first SVM is trained with the whole feature set consisting of all 232 features. The second SVM is trained on 62 selected features, the third one on 36 selected features, and the last SVM is trained on just 19 selected features. The y-axes depict the boxplots of the performance measures. The lower boxplot is generated from the accuracy values of the tested classifiers and the middle as well as the upper boxplots show the precision and recall rates of the class star crack, respectively. These plots reveal that the performances of all feature sets are comparable. Hence, we will use the quadratic SVMs trained on the whole feature set and on the selected 19 features for the performance comparison on the test set.

### 3.3. CNN Features and SVM

**Figure 12:**
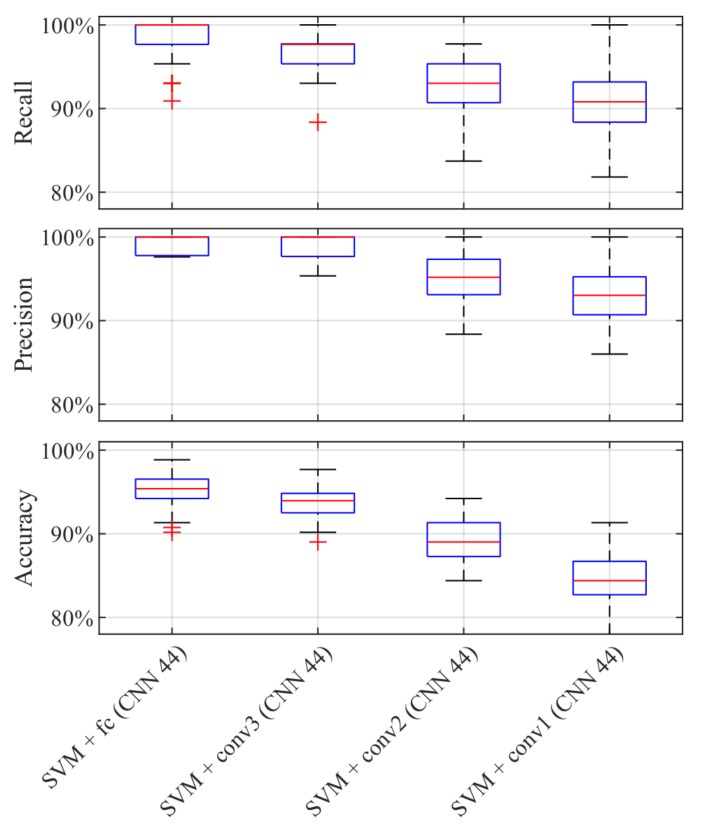
10-by-10 cross-validation results of SVMs trained on different ‘CNN 44’ features.

We extract the features learned by the simple CNNs of different layers and train quadratic SVMs. We perform a 10-by-10 repeated cross-validation for each feature set (Figure 12). The x-axes show the tested quadratic SVMs with the features extracted from the different network layers. The first SVM is trained on the features extracted from the fully connected layer. The second, the third, and the last SVM are trained on features extracted from the third, the second, and the first convolutional layer, respectively. The y-axes depict the boxplots of the performance measures. The lower boxplot is generated from the accuracy values of the tested classifiers and the middle as well as the upper boxplots show the precision and recall rates of the class star crack, respectively. These plots reveal that the performances of the first two SVMs have a comparable good performance. Hence, we will continue with comparing SVMs trained on the features extracted from the fully connected as well as the third convolutional layer on the selected simple CNNs yielding best overall accuracy, best precision, best recall, and best compromise between precision and recall.

**Figure 13:**
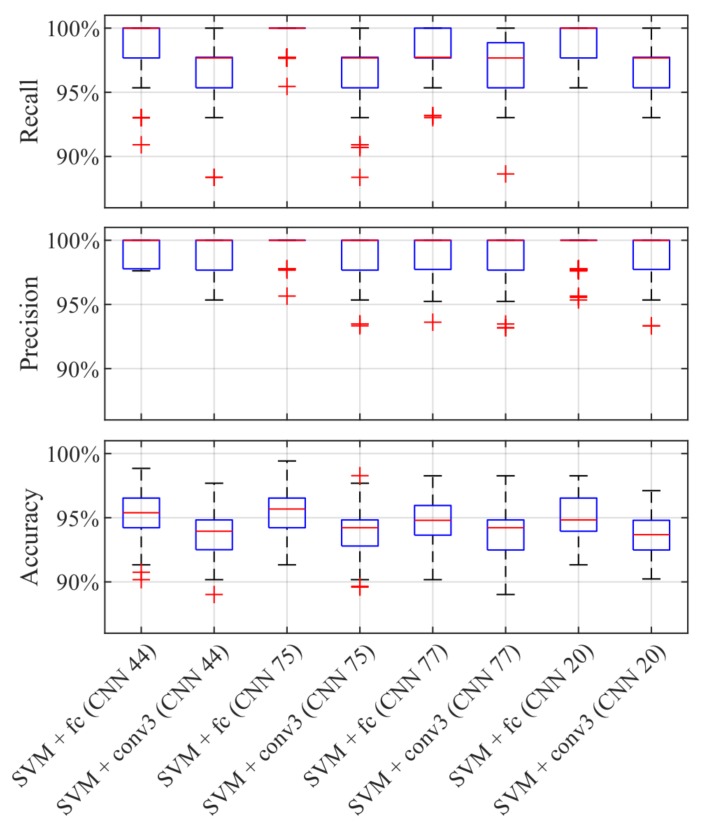
10-by-10 cross-validation results of SVMs trained on features extracted from the fully connected layer and the third convolutional layer of our selected trained simple CNNs.

Hence, we continue with evaluating these last two feature layers of our four selected simple CNNs. Figure 13 shows the 10-by-10 repeated cross-validation results of the quadratic SVMs trained on features extracted from the fully connected layer and the third convolutional layer of our selected trained simple CNNs. The first two SVMs are trained on features extracted from ‘CNN 44’, which is the trained simple CNN yielding the best overall accuracy. The second two SVMs are trained on features extracted from ‘CNN 75’, the one with the best precision. The third two SVMs are trained on features of ‘CNN 77’, the one achieving the best recall. The fourth two SVMs are trained on features of ‘CNN 20’, this is the net with the best compromise between precision and recall of the class star crack. This analysis shows that all trained SVMs are comparable regarding their performance.

### 3.4. Comparison: CNN vs. SVM vs. CNN + SVM

**Figure 14:**
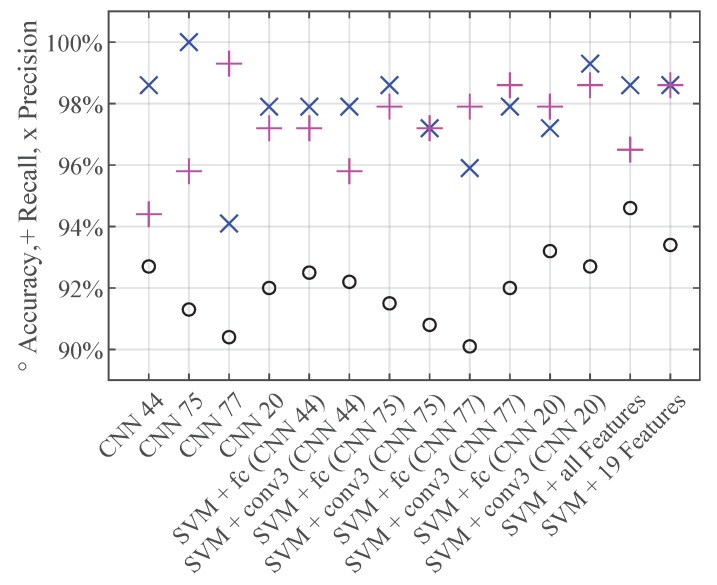
Performances of the models applied on the test samples.

Finally, we compare the performances of our classifiers on the test samples. These samples were not used to train the SVMs, nor to update the network weights of the CNNs. We apply the trained models on the test set and analyze their classification performances regarding their overall accuracy values and precision and recall rates of the class star crack (Figure 14). These performance measures are plotted on the y-axis. The x-axis shows the different trained models. This plot allows us to draw four conclusions. The first one is that all classifiers are performing well yielding overall accuracy values above 90%. The second one is that the first three CNNs have a difference of around 4% between their recall and precision values. However, if we extract just the features of these CNNs and classify the test samples with an SVM, the resulting precision and recall values are about the same value. The third observation is that the SVM trained on the features extracted from the third convolutional layer of the ‘CNN 20’ has the best performance on the test samples. It reaches a good overall accuracy and a high precision and recall rate for the class star crack. The ‘CNN’ 20 is the net with the best compromise between precision and recall. The fourth conclusion is that the results of the quadratic support vector machine trained with the 19 selected features of the manual feature extraction is comparable to the one trained with the features of the third convolutional layer of ‘CNN 20’.

**Figure 15:**
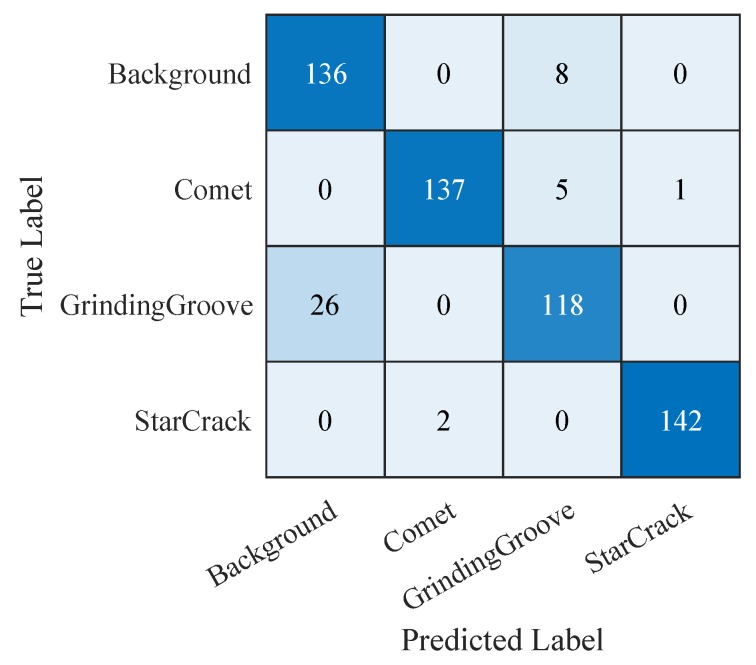
Confusion matrix of ‘SVM + conv3 (CNN 20)’ applied on the test samples.

**Figure 16:**
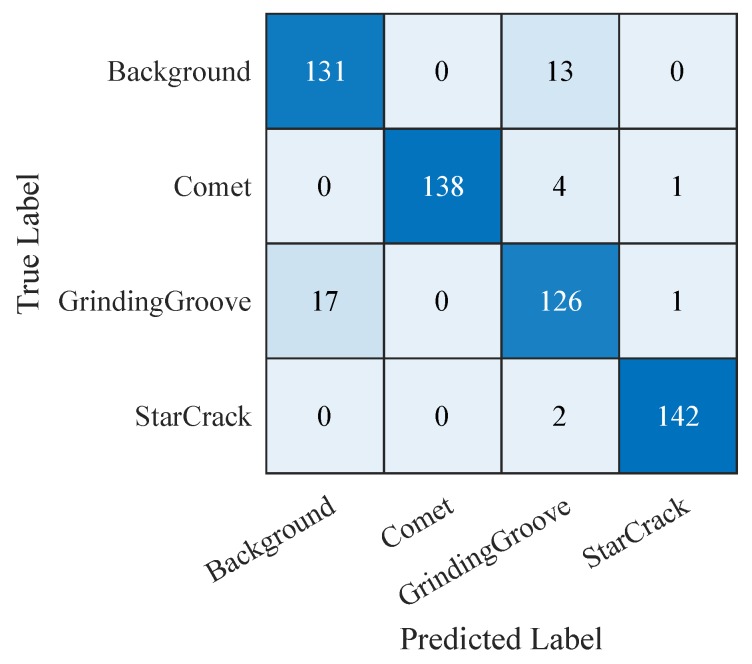
Confusion matrix of ‘SVM + 19 Features’ applied on the test samples.

**Figure 17:**
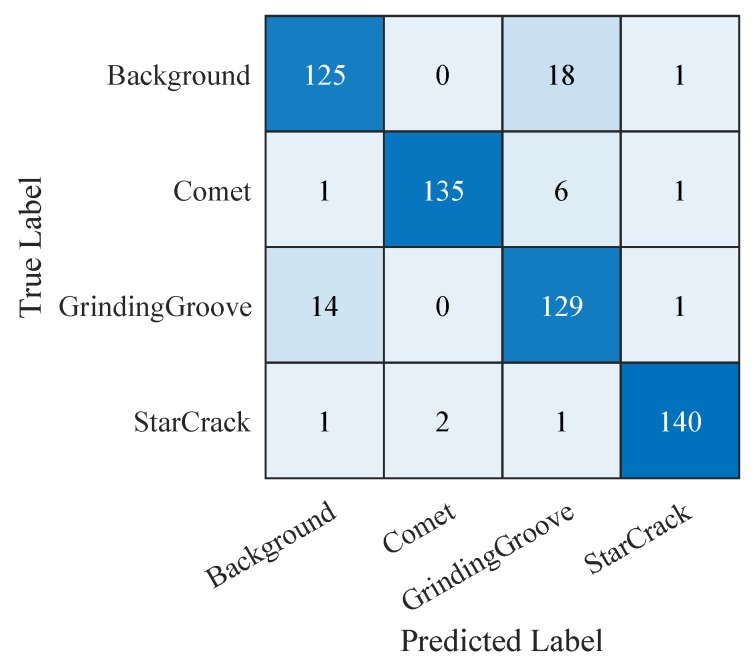
Confusion matrix of ‘CNN 20’ applied on the test samples.

Hence, we continue with comparing the confusion matrices of the SVM trained on the features extracted from the third convolutional layer of the ‘CNN 20’ (Figure 15), the SVM trained on the 19 selected features of the manual feature extraction (Figure 16), and the ‘CNN 20’ (Figure 17). The confusion matrices plot the predicted labels against the true labels. The predicted labels are plotted on the x-axes and the true labels on the y-axes. All correctly classified samples are listed in the diagonal fields from the top left corner to the bottom right corner. All the other fields contain wrongly classified samples. By analyzing them we observe that the classes grinding groove and background get more confused than the other ones. The reason therefore is that their signals in the input images are more difficult to distinguish compared to the other ones. However, we focus on correctly classified star cracks in this work. Therefore, we concentrate on the bottom right corners as well as on the last rows and the last columns. The bottom right corners display the number of correctly classified star cracks. The numbers of star cracks wrongly classified as background, comet, or grinding groove regions are depicted in the last rows. The last columns display the background, comet, and grinding groove regions wrongly classified as star cracks. Additionally, we consider the overall accuracy values of all three classifiers.

Figure 15 shows the confusion matrix of the ‘SVM + conv3 (CNN 20)’ applied on our test samples. This SVM is trained on the features extracted from the third convolutional layer of the ‘CNN 20’. It correctly classifies 142 star cracks out of 144. Two star cracks are wrongly classified as comets. This results in a recall rate of 98.6% of the class star crack. The regarding precision rate of the class star crack is 99.3%. Only one comet is wrongly classified as a star crack. The overall accuracy is 92.7%.

Figure 16 shows the confusion matrix of the ‘SVM + 19 Features’ applied on our test samples. This SVM is trained on the 19 selected features of the manually extracted features. It also correctly classifies 142 star cracks out of 144. In this case, two star cracks are wrongly classified as grinding grooves. Hence, this results in a recall rate of 98.6% of the class star crack too. The regarding precision rate of the class star crack is also 98.6%. Here, one comet and one grinding groove region are wrongly classified as a star crack. The overall accuracy is 93.4%.

Figure 17 shows the confusion matrix of ‘CNN 20’ applied on our test samples. It is the trained simple convolutional neural network with the best compromise between recall and precision of the class star crack. This CNN also correctly classifies 140 star cracks out of 144. Here, one star crack is wrongly classified as background region, two star cracks are wrongly classified as comets, and one star crack is wrongly classified as a grinding groove region. Hence, this results in a recall rate of 97.2% of the class star crack. The regarding precision rate of the class star crack is also 97.9%. Here, one background region, one comet, and one grinding groove region are wrongly classified as a star crack. The overall accuracy is 92.0%.

**Figure 18:**
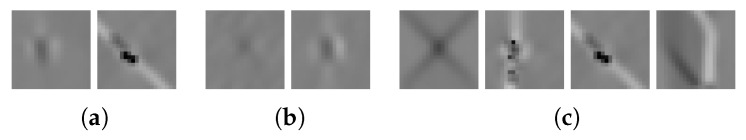
(**a**) Star cracks predicted as comets by SVM + conv3 (CNN 20), (**b**) star cracks predicted as grinding groove regions by SVM + 19 Features, (**c**) one star crack predicted as background region, two star cracks predicted as comets, and one star crack predicted as grinding groove region by CNN 20.

Figure 18 shows the topography images of all star crack image stacks of the test set, which are wrongly classified as comets, grinding grooves, or background regions. Analyzing these samples allows us to draw two conclusions. The first is that there is some overlap between weak signals of star cracks and signals of comets or grinding grooves (Figure 18a,b). The second conclusion is that the CNN model misses a typical star crack and predicts it as a background region (Figure 18c, first image).

**Figure 19:**
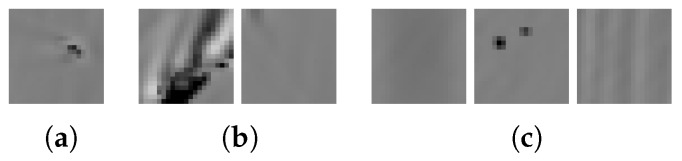
(**a**) One comet predicted as star crack by SVM + conv3 (CNN 20), (**b**) one comet and one grinding groove region predicted as star crack by SVM + 19 Features, (**c**) one comet, one grinding groove region, and one background region predicted as star crack by CNN 20.

Figure 19 shows the topography images of all test set image stacks wrongly classified as a star crack. Analyzing these samples allows us to draw two conclusions. The first one is that there is an overlap between signals of grinding groove regions and weak star cracks. The second one is that strong comet signals are predicted as star cracks.

**Figure 20:**
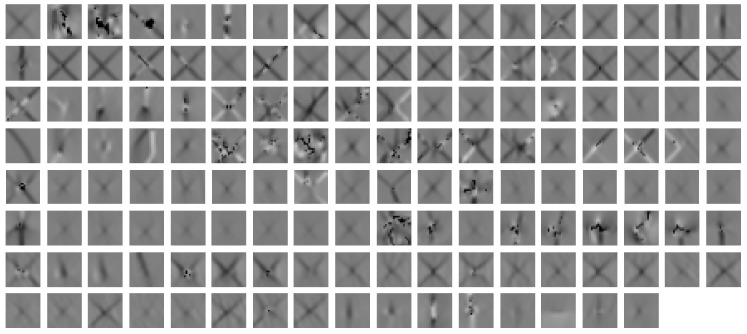
Image stacks correctly predicted as star cracks by SVM + conv3 (CNN 20) as an example.

Figure 20 shows the topography images of all correctly classified star cracks by the ‘SVM + conv3 (CNN 20)’ as an example. The ‘SVM + conv3 (CNN 20)’ is the quadratic SVM trained on the features extracted from the third convolutional layer of the ‘CNN 20’. This illustrates how good the trained model can deal with different sizes, shapes, and contrasts of star cracks.

Finally, we compare the times elapsed during testing on a single i5 CPU using MATLAB [28]. The ‘CNN 20’ only needs around 1 second to classify the test set. The SVM trained on the features extracted from the third convolutional layer of the ‘CNN 20’ has a processing time of around 2 seconds for classifying the test set. The SVM trained on the 19 selected features of the manual feature extraction requires the longest processing time of around 15 seconds for classifying the test set, due to the manual feature extraction. Overall, the processing time for new samples is sufficiently low for all three models.

## 4. Conclusions

In this work we classified image stacks of silicon wafer back surfaces with three different approaches: CNNs, SVMs trained on manually extracted features, and SVMs trained on features extracted by CNNs. The image stacks of the wafer back surfaces are divided into four classes: Background, comet, grinding groove, and star cracks. Detecting image stacks containing star cracks was the goal of this work. We achieved an overall testing accuracy above 90% with all three approaches. Focusing on the class star crack also showed good results with over 97% precision and recall rates with all three approaches. The best compromise between precision and recall rate of the class star crack could be achieved with the simple convolutional neural network architecture used as a feature extractor followed by a quadratic support vector machine. It reached a precision rate of 99.3% and a recall rate of 98.6% on the test set. However, we must be mindful that we chose the best performing convolutional neural net out of the hundred trained nets for these comparisons. If the training time allows us to train the convolutional neural net only once, chances are that the results of the approaches involving the convolutional neural nets are worse compared to the statistical approach.
